# The Limits of Mean-Field Heterozygosity Estimates under Spatial Extension in Simulated Plant Populations

**DOI:** 10.1371/journal.pone.0043254

**Published:** 2012-08-27

**Authors:** James L. Kitchen, Robin G. Allaby

**Affiliations:** School of Life Sciences, University of Warwick, Coventry, United Kingdom; Biodiversity Insitute of Ontario - University of Guelph, Canada

## Abstract

Computational models of evolutionary processes are increasingly required to incorporate multiple and diverse sources of data. A popular feature to include in population genetics models is spatial extension, which reflects more accurately natural populations than does a mean field approach. However, such models necessarily violate the mean field assumptions of classical population genetics, as do natural populations in the real world. Recently, it has been questioned whether classical approaches are truly applicable to the real world. Individual based models (IBM) are a powerful and versatile approach to achieve integration in models. In this study an IBM was used to examine how populations of plants deviate from classical expectations under spatial extension. Populations of plants that used three different mating strategies were placed in a range of arena sizes giving crowded to sparse occupation densities. Using a measure of population density, the pollen communication distance (*P_cd_*), the deviation exhibited by outbreeding populations differed from classical mean field expectations by less than 5% when *P_cd_* was less than 1, and over this threshold value the deviation significantly increased. Populations with an intermediate mating strategy did not have such a threshold and deviated directly with increasing isolation between individuals. Populations with a selfing strategy were influenced more by the mating strategy than by increased isolation. In all cases pollen dispersal was more influential than seed dispersal. The IBM model showed that mean field calculations can be reasonably applied to natural outbreeding plant populations that occur at a density in which individuals are less than the average pollen dispersal distance from their neighbors.

## Introduction

Understanding the evolutionary process is increasingly requiring in the integration of sources of data that are typically beyond classical population genetics models [1]. One such example is the inclusion of spatial extension. Classical population genetics approaches typically use a simplified mean-field approach in which individuals of a population or subpopulation conceptually occupy the same space, and are therefore subject to the same conditions and pressures, and purely random mating occurs. Along with other simplifying assumptions such as non-overlapping generations and constant population sizes, such populations behave in tractable ways that can be described through deterministic approaches leading to features such as Hardy-Weinberg equilibria [2] and the Wright-Fisher model [3]. The predictions of such models provide a useful starting point for evolutionary studies, for instance in establishing whether there has been significant deviation from neutrality indicative of selection. However, Mayr [4] observed that it was surprising how little classical population genetics has contributed to the understanding of one of the most important processes in evolution, speciation. This is because a mean-field based model is essentially based on an anagenic evolutionary system, rather than a cladogenic one [5]. The evolutionary differentiation of populations, which ultimately leads to speciation, requires different selective environments.

In reality populations are spread across environments that are often patchy with different sets of conditions. Environmental conditions may have a variety of effects, such as impeding dispersal [6] or providing different selective niches [7]. Plant mates are unlikely to be entirely chosen at random. For instance, plants have been known to have assortative mating due to pollinator foraging behaviour regarding petal color [8], inflorescence height [9] and overlapping flowering times [10]. Furthermore the spatial dimension is of particular importance even within a single constant environment because of their sedentary nature causing a bias for nearest-neighbor mating over panmixia [11]. The spatial density of a plant species affects the optimal mating strategy with higher out-crossing rates being more suited to higher densities [11] and selfing systems evolving to invade low-density environments [12]. The boundaries and margins between contrasting selection environments may be areas in which globally rare but locally abundant alleles occur through gene flow between areas in which they have high selective value to areas of low or neutral selective value, giving rise to processes such as the persistence of unfit alleles which would not be detected in mean field based model systems [1]. A problem with spatially extended plant populations in nature recently highlighted is that it is unclear to what extent classical population genetics tools can meaningfully be applied at all when individuals are continuously distributed across space [13].

It is therefore desirable to include the dimension of spatial extension into population genetics models in order to effectively incorporate these factors and more accurately reflect the evolutionary process. Individual-based modeling (IBM) is a versatile approach for the integration of non-parametric and complex factors into population genetics. Spatial extension can readily be incorporated into IBM and has been successfully applied to a variety of problems including plant domestication [14], [15], predator prey relationships [16]–[20] and the emergent field of landscape genetics [21]–[23]. As ecological boundaries, environmental conditions and varying dispersal distances can be readily incorporated into these models they have been used to study speciation events [24]–[26] and hybrid zone formation [27]. Furthermore as these models readily allow individual behaviors to be defined they have been useful for simulating gender specific mating preferences and the resultant effects of assortative mating on speciation and hybridization [28]–[29]. Cuddington and Yodzis [16] demonstrated that there is a reduced mobility in spatially explicit relative to mean-field models, resulting in reduced reproduction rates in the former. By reducing mobility parameters of the mean-field model, a close congruence between the two model types was achieved. The differentiating effects of spatial extension are likely to increase as individuals become more distantly spaced out. However, it is still unclear how the relationship between the two systems changes with increasing spatial extension.

We have developed an IBM to study the evolution of plant systems in order to integrate multiple diverse data sources and structures [1]. In brief, the IBM is implemented as follows: diploid individuals are arranged onto a two-dimensional grid made up of cells each of which can hold a single adult plant. The individuals contain genes that can accumulate neutral mutations and gametes are generated by Mendelian segregation of homologous chromosomes. The simulation updates in intervals each representing one month. Individuals begin as seeds and may germinate with a defined probability on condition of the absence of an adult in the cell. The individuals then move through the following life cycle stages: seed, vegetative growth, flowering and senescence, with the duration of each life cycle stage varying between individuals. Flowering individuals disperse pollen and seeds and may self or out-cross according to a user-input probability. Individuals senesce and are removed a number of updates after dispersing their seeds determined by randomly sampling.

In this study we aimed to determine under what conditions, if any, parameters estimated by classical population genetics approaches can be applied approximately to establish the presence of conditions of equilibria and neutrality in a spatially extended system. The IBM was used to generate plant populations of varying spatial densities, mating systems, and pollen and seed dispersal capabilities. We assessed the extent of deviation between classical population genetics and the spatially extended system by comparing values of the heterozygosity parameter. Heterozygosity was measured as expected from classical population genetics through the Hardy-Weinberg law (*H*_e_), and compared to the real value obtained from individuals (*H_o_*). As far as we are aware this is the first attempt to determine how perturbations of spatial extension affect the accuracy of mean-field based calculations of population parameters.

## Results

**Figure 1:**
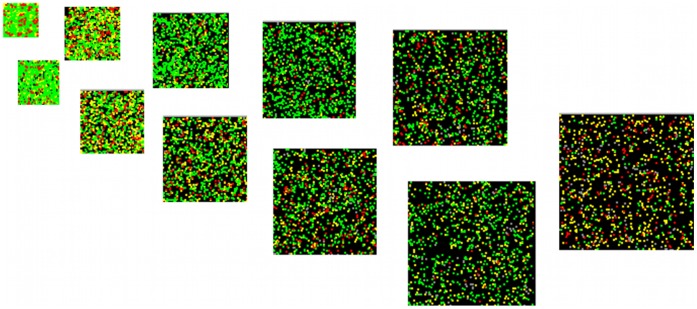
Model screen shots of the different matrix sizes used in the study. The void cells are in black with grey cells representing unoccupied non-void cells. The different colors of the individuals represent their different life-cycle stages, with red = seeds, green = vegetative and yellow = flowering/senescence.

All simulations in this study began with populations of 1000 individuals in a two dimensional matrix with *M* cells, in which a single cell could contain a single growing plant. Simulations were carried out for 4000 updates with a burn-in period in which populations were allowed to equilibrate (see methods). For each set of conditions, simulations were repeated ten times. To prevent individuals from forming clusters with each other [30], we used a strategy in which cells were randomly blocked out (void cells) within all matrices from all simulations, such that they could not be occupied until just 1000 cells remained. On average this achieved an even spacing between individuals in our simulations with a greater degree of spacing between individuals within the larger matrices, Figure 1. In preliminary simulations where blocking was not applied we observed clustering behaviour even within larger matrices (data not shown).

### Mating System

**Figure 2:**
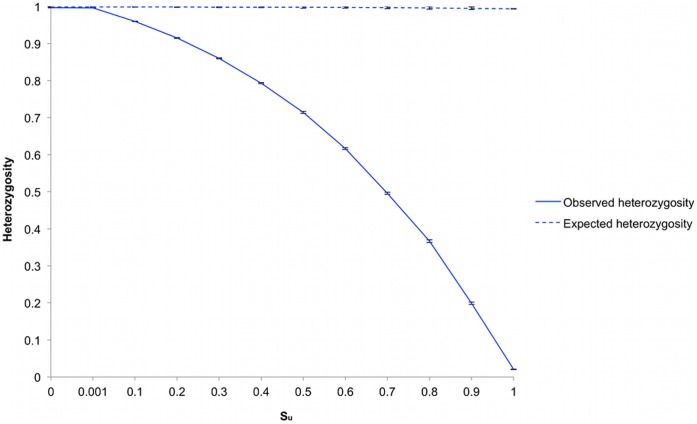
Heterozygosity as a function of selfing probability. H_o_ is plotted with the solid line and H_e_ using a dashed line. Simulations were repeated ten times. Error bars represent the standard error.

The first set of simulations investigated the effect of mating system in a spatially explicit population. In this case a matrix size of 35^2^ cells was used in which 225 cells were randomly blocked. In this matrix size most individuals were immediately adjacent to other individuals in all eight surrounding cells. The model takes a user input of the probability of self-pollination, *S_u_,* to allow control over the outcrossing exhibited by flowering individuals. The value *S_u_* is the probability that a particular pollen grain will self-fertilize the parent plant rather than leave the flower and pollinate another plant. Simulations were carried out with *S_u_* ranging in value from 0 to 1, Figure 2. At values of *S_u_* at 0 and 0.001, the resulting values of *H_o_* and *H*_e_ overlapped, and at a *S_u_* of 0.1 were within 5% of each other suggesting *H_o_* gave a good approximation of *H_e_*. *S_u_* equal to 0 is representative of a self-incompatible mating strategy, while a value of 0.001 represents a level of out-crossing equal to that found in panmictic systems. Increasing values of *S_u_* in the spatially extended system resulted in a reduction in heterozygosity, as would also be expected of a mean field system [31].

### Matrix Size

**Figure 3:**
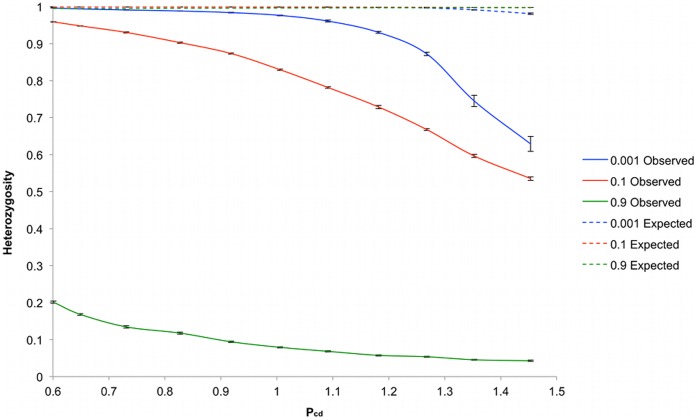
Heterozygosity as a function of matrix sizes. Heterozygosity was plotted at S_u_ = 0.001 (blue), S_u_ = 0.1 (red) and S_u_ = 0.9 (green). H_o_ is plotted with the solid line and H_e_ using a dashed line. Simulations were repeated ten times for each value of S_u_, with error bars representing the standard error.

We investigated the effect of increased separation between individuals in a spatially extended population. Simulations were carried out with progressively increased matrix sizes ranging from 35^2^ to 135^2^ cells, with increments of 10 in both matrix dimensions between each set of simulation conditions, Figure 1. Each simulation was performed with M - 1000 void cells, allowing the population size to remain constant between different sets of simulation conditions. Three series of simulation experiments were carried out with different mating strategies defined by values of *S_u_* of 0.001, 0.1 and 0.9, respectively, Figure 3. We used the parameter pollen communicating distance (*P_cd_*) to express the degree of separation between individuals defined as:
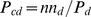
(1)Where *nn_d_* is the average distance to the nearest neighbor in matrix cells, and *p_d_* is the average dispersal distance of pollen in matrix cells. We also measured a second parameter, the seed communicating distance (*S_cd_*) which is defined as:
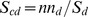
(2)Where 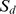 is the average dispersal distance of seeds in matrix cells. Constant values of maximum dispersal of *Sd* and *Pd* were used of 10 cells.

The population size per update in simulations with values of *M* over 13225 and *S_u_* 0.001 was often low, leading to an increased variation of *H_o_* which ranged in standard deviation 0.015–0.064. At smaller matrix sizes the standard deviation ranged 4×10^−4^−0.008. This increase in variation was probably due to high mortality and low fertility rates under these conditions in the model resulting from low numbers of nearby vacant cells for seeds to disperse to, and low numbers of nearby pollinating individuals (not shown). *Ho* decreased as the *P_cd_* increased with matrix size. The relationship between *P_cd_* and *H_o_* in Figure 3 varied between the three mating systems tested. In the case of *S_u_* equal to 0.001, the rate at which *H_o_* decreased with increased *P_cd_*, or *r,* as we shall use in the text, is given by the gradient of the line, which varies either side of a *P_cd_* value of 1.1. At values of *P_cd_* lower than 1.1 *r* is relatively shallow at [−0.005], and steepens considerably to [−0.103] at *P_cd_* values higher than 1.1. In contrast, in simulations where *S_u_* was equal to 0.1, an approximately linear relationship occurred between *H_o_* and P_cd_ in which *r* was relatively constant. At the highest values of *P_cd_* these two sets of simulations produced similar values of *H_o_.* In the third set of simulations where *S_u_* was equal to 0.9, values of *H_o_* decreased to an asymptote.

The *S_cd_* parameter describes the movement of seeds in relation to the spatial density of plants, and we expected it to have some influence on heterozygosity also. At the minimum matrix size a *S_cd_* of 0.9 occurred, while at maximum matrix size this value was 2.2. Therefore, in the smallest matrix system the dispersal ranges of seeds encompassed neighboring individuals, but not in the case of the largest matrix suggesting that seed movement was contributing to gene flow in the former, but much less in the latter.

A threshold effect is apparent in these results, in which under a panmictic mating system deviation from the classical expectation is small, typically within 5%, while plants are within a range of their nearest neighbor, with an average nearest neighbor distance less than the average pollen dispersal distance. After this threshold the deviation from classical expectations increases rapidly with increasing separation. However, a more mixed mating strategy shows a steady deviation from classical expectation with increasing matrix size indicating that spatial separation has a more direct effect in these cases. Interestingly, these two sets of simulations reach similar levels of heterozygosity at the maximum extent of separation investigated in this study. The isolation caused by the highly selfing system has a large effect on heterozygosity even in small matrices with low *P_cd_* values, which then decreases to a minimum equilibrium which is probably kept from reaching zero by the influx of new mutations. In these cases, the selfing mating strategy is the principal influence on heterozygosity.

### Pollen and Seed Dispersal

**Figure 4:**
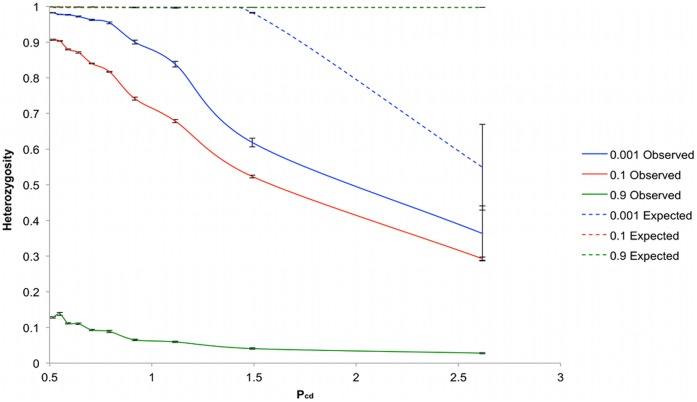
Heterozygosity as a function of P_cd_. Heterozygosity was plotted at S_u_ = 0.001 (blue), S_u_ = 0.1 (red) and S_u_ = 0.9 (green). H_o_ is plotted with the solid line and H_e_ using a dashed line. Simulations were repeated ten times for each value of S_u_, with error bars representing the standard error.

**Figure 5:**
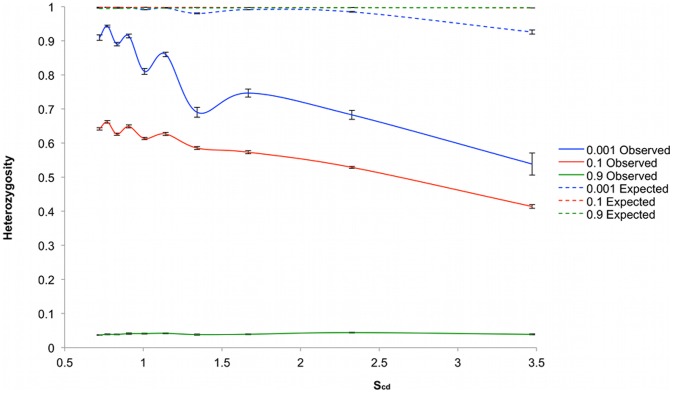
Heterozygosity as a function of S_cd_. Heterozygosity was plotted at S_u_ = 0.001 (blue), S_u_ = 0.1 (red) and S_u_ = 0.9 (green). H_o_ is plotted with the solid line and H_e_ using a dashed line. Simulations were repeated ten times for each value of S_u_, with error bars representing the standard error.

An alternative to increasing the space between individuals to increase spatial isolation is to reduce dispersal of pollen and seeds. If there are no other influencing factors introduced by increased spacing of individuals, then altering the dispersal behavior should allow similar levels of heterozygosity to be achieved under differentially extended systems. We tested this prediction by performing simulations at the largest matrix size (*M* = 135^2^) in which we progressively increased pollen (Figure 4) or seed dispersal (Figure 5). Simulations were performed under the three different mating systems of *S_u_* equal to 0.001, 0.1 and 0.9. We used the parameter *P_cd_* to measure extent of separation of individuals expressed through pollen communication, and the equivalent parameter *S_cd_* to express the extent of movement of seeds.

A threshold effect was again apparent in the pollen dispersal based perturbation simulations when *S_u_* was set to 0.001, in which the gradient of the line varied either side of a *P_cd_* value of 0.9, from −0.005 to −0.183, Figure 4. Generally, lower values of *H_o_* at each *P_cd_* were obtained in this simulation than in the matrix size simulations for the same mating system. This is likely to be due in part at least to the effects of seed dispersal. The value of *S_cd_* in the pollen dispersal based perturbations was consistently 2.3. However, *S_cd_* varied in the matrix size based perturbation simulations from 0.9–2.2. Consequently, at low values of *P_cd_* there was a disparity in the effect of seed dispersal between the two systems, and the higher *H_o_* of the matrix size perturbation based simulations is explicable due to the greater gene flow mediated by seed movement. The remaining two mating system simulations also produced results that echoed those of the matrix size based perturbation simulations (Figure 3), but with consistently lower values of *H_o_*.

The effect of increasing seed dispersal had a more limited effect on increasing *H_o_* than that observed with pollen dispersal, Figure 5. In these simulations, the *P_cd_* value was consistently 1.5. In the case of simulations with *S_u_* of 0.001, the results had a great deal of variance leading to a jagged line plotted of *H_o_* against *S_cd_*. In this case the jagged line correlates closely with the average population sizes generated in the simulations that varied considerably and included very low sizes (less than 100 individuals, data not shown). This is because low population densities (as defined by high values of *P_cd_* and *S_cd_*) are subject to a high risk of failure to reproduce under this mating system, which requires 99.9% outbreeding, because there may be an inadequate supply of neighbors sufficiently close for pollination. The other mating system simulations where *S_u_* was 0.1 and 0.9 did not give rise to such an erratic set of results.

In all simulations the effect of increasing the seed dispersal on *H_o_* was less than increasing the extent of pollen dispersal that demonstrates that although increasing seed dispersal has an influence on heterozygosity, it is weaker than the effect of pollen flow. Furthermore, we did not observe a clear threshold effect for seed dispersal.

## Discussion

To our knowledge this is the only study where increasing spatial perturbations are made to examine the discrepancy between real observed values of population genetics variables, and those expected based on mean-field population genetics theory. The results we present here demonstrate that the deviation from classical population genetics introduced by the closer approximation to the real world through spatial extension is largely tractable, and different for different mating systems. We suggest that extent of spatial extension can be usefully viewed through a concept of effective population density which is described in the *P_cd_* and *S_cd_* variables, which can be measured directly for any plant species population in nature.

Our findings suggest that in outbreeding populations systems in which the *P_cd_* and *S_cd_* values are below 1, the deviation from classical expectations is generally low (less than 5%), and therefore classical population genetics approaches may make acceptable approximations of reality. The approximation breaks down rapidly once the threshold value of ∼1 is exceeded for *P_cd_* in particular. In the case of inbreeding systems, typical of sparsely populated systems, the effects of inbreeding are far greater than any introduced by spatial extension, consequently mean-field based adjustments for inbreeding may also be adequate in a spatially extended system. However, we found that populations with mixed mating strategies are more sensitive to increased spatial extension, and in these cases values generated from classical population genetics are likely to be poor approximations of the true values.

In order to study the effects of spatial extension we prevented clumping from occurring, which has been observed in previous studies [30], It may be argued that in reality populations form a clumpy pattern of distribution. We found that increasing matrix size and nearest-neighbor distances had little effect on lowering *Ho* when individuals were clumped together (not shown). Therefore clumping in this case may confound the effects of spatiality, even though clumped subpopulations maintain a spatial population structure. When we Increased seed and pollen dispersal on clumped individuals, however, we saw similar increases in Ho as with spaced individuals, consistent with previous observations [30]. We suggest that clumped populations in the real world could usefully be considered within the effective population density framework presented here. Values of *P_cd_* and *S_cd_* could be considered within and between clumps. Often, these values of *P_cd_ and S_cd_* within clumps would be low such that classical population genetics could be applied as an approximation. The *P_cd_* and *S_cd_* values between clumps may well be high enough to identify the expectation of genetic structure in the population, This in part answers Platt et al’s [13] concern, that a lack of geographic barriers and continual changes in spatial genetic autocorrelation observed in *Arabidopsis thaliana* suggests that no single population structure could be identifiable, and therefore that application of classical population genetics could be problematic.

Our simulated results show that under certain conditions spatially extended populations closely approximate HWE, and this is verified by studies of plant populations in the real world [34]–[37]. These studies include plants with various pollen and dispersal systems, including both abiotic and biotic mechanisms. Plant populations of Sandalwood, for instance, with high dispersal and outcrossing rates conform to HWE [38] are consistent with our simulated results. In our simulations we observed that population sparcity leads to deviations from HWE. This deviation has also been observed in reality with sparse plant populations in species of juniper and poppy [39]–[40]. Conversely, it is expected that higher outcrossing frequencies should be associated with higher population densities, and so a tendency to conform to HWE, and this too has been observed in wind-pollinated conifers [41] and in *Mimulus ringens* [42]. Our simulations predict that some plant populations would be observed to conform to HWE in some cases and deviate from it in other cases due to the consequences of limited dispersal relative to stand density. This finding is concordant with that of Dering and Chybicki [43] who compared the genetic diversity of natural and artificial regenerations of *Quercus robur* (L.) and *Quercus petrea* (Matt.) Liebl. populations. In this case the natural regenerations were in HWE, but the artificially regenerated progeny plantations were not because of limitations in dispersal caused by the sowing regime. Similarly, limitations of dispersal caused deviations from HWE in 1 out of 14 sampled populations of *Striga hermonthica* [44]. Our simulations further showed that HWE is restored in sparse populations where dispersal rates are increased to rates in which values of *P_cd_* are 1 or less. This effect is observed in *Desmodium nudiflorum*, which despite apparent population sparcity has long-range seed dispersal by animals [45].

This study demonstrates that our IBM system behaves as expected under neutral conditions, and has given some useful insight into how the relationship between stand density and dispersal as encompassed by the *P_cd_* and *S_cd_* values is a predictor of adherence to HWE that could be applied to the real world. The model therefore has utility in exploring hypotheses in which deviation from neutral expectations occurs due to other factors, such as selection. An emergent observation from studies of evolution at the systems and genomic level is that simple scenarios that affect single genes are rare, and that often many genes and regulatory networks are involved, as observed with humans [46]–[47]. Similarly, with the evolution of domesticated plants estimates of the number of genes underlying the domestication syndrome traits range from 27 to 70 in wheat [48]–[49]. Therefore there is a need to be able to consider evolutionary change in a way that connects the interdependency of gene networks, genome architecture to spatially explicit populations. The IBM approach presented here is designed to achieve this end by having individuals that are capable of supporting gene networks in a virtual genome, such that a systems biology architecture can be connected to a spatially explicit population level of organization and selection. We believe that such approaches will enable the computational exploration of evolutionary genetics to move to a new level in which systems approaches and spatially explicit population genetics are integrated.

## Methods

### Model

Individuals are arranged onto a two-dimensional grid-like matrix. Cells may be occupied by a plant, or be empty. In each update the model processes each individual by iterating its age and allowing it to interact with other individuals. Each grid cell may contain only one adult and ten seeds. A ‘seed queue’ system was implemented to replace older seeds with newer seeds and discard the older seeds. Each simulation begins with 1000 diploid individuals each containing two genes per genome copy. When individuals germinate they can accumulate mutations, which are passed on to their progeny. Gametes are produced by Mendelian segregation of the individual’s genes at the floral stage of the life cycle. Individuals will then either pollinate themselves or other flowering individuals and then disperse their resultant seeds. Once individuals have dispersed their seeds they senesce and are removed from the model.

#### Burn-in

To ensure there had already been an accumulation of mutations as the simulations start, a burn-in algorithm was implemented. For each of the 1000 starting individuals 4000 updates with mutations occur to the genes at each copy of the genome, according to the input mutation rate of 10^−4^. The resulting genotypes are then randomly assigned to each of the starting individuals.

#### Individuals

Each individual has the following life cycle stages: seed, vegetative, flowering and senescence. The number of updates for an individual to move to each life stage is drawn from a Poisson distribution. Once a seed has germinated it is considered to be an adult. Individuals may disperse pollen and reproduce within their flowering stage, with the number of ovules per individual set to 30. At the end of the flowering life stage all fertilized ovules are dispersed as seeds. At the end of the senescing life stage the individual dies and is removed from the model.

#### Matrix

Simulations were run with increasing matrix sizes of 35^2^ cells to 135^2^ cells in steps of 10 cells in each dimension. In each simulation, cells were made unoccupiable in the matrix thus preventing individuals from occupying these cells, termed ‘void cells’. The number of void cells per simulation was calculated according to the formula:
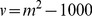
(3)where *v* is the number of void cells and m^2^ is the matrix size in cells. Simulations for each matrix size were repeated ten times. The matrix was constructed using a quadtree data structure [32]: this is a tree-like structure where objects are stored in two-dimensional space, with the whole matrix being the root node. As objects are added to the tree the tree nodes are recursively split into four more nodes, until the smallest node size has been reached (which was set to a 2×2 sub-matrix). When an individual is extracted for distance calculations the tree nodes are recursively searched for the specified (*x,y)* coordinates of the individual until the smallest node containing the individual is obtained. Groups of individuals are extracted in a similar way using a radius. This method provided better performance than using an exhaustive search approach.

#### Dispersal

In this model pollen is dispersed by wind. A probability for self-fertilization per ovule, S_u_ is input by the user, if this probability value is overcome then the ovule may be pollinated by an outcrossing event. Seeds and pollen are dispersed using a cumulative form of the function, described in [33] for wind-pollinated plants such that the probability of a pollen grain travelling *j* cells is given by:
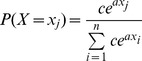
(4)Where *x_j_* is a distance in cells that a pollen grain is dispersed. The maximum number of cells pollen (*P_max_*) can disperse is given by *n*. The variable c is the intercept and *a* is the gradient of the underlying function:



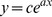
(5)A value of 1.5 was used for *c*. For pollen *x* is considered in the range *1≤ x ≤P_max_*.The form of the equation used for seeds is similar, except that *x* is considered in the range *0≤ x ≤ S_max_* These variations ensure that pollen exiting the flower does outcross but that seeds can fall onto the same cell as their parent individual. *P_max_* and *S_max_* were user input. For pollen, *a* was calculated as:
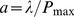
(6)Where λ is a constant set to −2 for pollen dispersal. An equivalent equation is used for seed dispersal, with the denominator set to S_max_ and λ set to −4. A maximum dispersal distance for both seed and pollen is input as a user defined parameter. The distance (*D*) a pollen or seed was dispersed was determined by generating a random number in the range of 0 to 1 that was used to sample under the probability distribution generated from equation (4).

Once distance of dispersal had been determined, the direction of dispersal was generated. Two different approaches were used for pollen and seeds respectively. The direction of dispersal was assumed to be due to a prevailing wind. The general direction of the wind was randomized for each update. The angle of the prevailing wind in degrees, *θ,* is drawn from a uniform distribution each update and is relative to the vertical axis. The specific direction of dispersal is generated from *θ* by resampling from a normal distribution with mean *θ* and a standard deviation of 180° to produce *y*, the specific angle of dispersal. The destination cell is determined to which the seed will be dispersed using the values of *γ* and *x*.

For pollen dispersal, we began with the recipient of pollen and calculated the relative probabilities of pollination by all potential pollen donors. Potential pollen donors were those individuals that were within the range of the recipient defined by *P_max_*. For each donor, the angle (φ) was calculated which is defined as the angle of the recipient to the donor relative to a vertical axis passing through the donor. A quantile value (*q_i_*) was calculated for φ from the normal distribution of mean *θ* (angle of wind direction) and standard deviation of 15 (degrees), which gave a measure proportional to the probability of pollen emanating from the donor in the direction of the recipient. The probability of the donor *d_i_* being the successful pollen donor to the recipient was then calculated as:
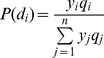
(7)Where *y_i_* is calculated as in equation (4) for the distance *x_i_* between potential donor and recipient, and *n* is the total number of potential pollen donors. The successful pollen donor was then selected through random sampling of all possible donors. The process was repeated independently for each ovule to be pollinated on the recipient plant.

### Analysis

#### Heterozygosity calculations

The observed heterozygosity, *H_o_* of remaining individuals after 4000 updates of each simulation was recorded and compared with the expected heterozygosity as calculated by an ideal population under Hardy-Weinberg equilibria, as in [23]. The Hardy-Weinberg formula for multiple alleles was used to calculate *H_e_*.

#### Average nearest neighbor distances

To calculate the nearest neighbor distance an individual plant *i*, the distance (D) to all other individuals was calculated based on their Cartesian coordinates using:
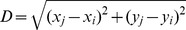
(8)Where *x_i_* and *y_i_* are the x and y coordinates of the individual *i*, and *x_j_* and *y_j_* are coordinates of the *j*th individual. The lowest value of *D* was recorded for each individual. All recorded *D* values were averaged at the end of the simulation to produce an average nearest neighbor distance For most simulations, the pollen dispersal distance (*P_d_*) or seed dispersal distance (*S_d_*) was recorded for each dispersal event in number of cells. The effect of the dispersal is context dependent on the density of individuals in the population, so we used an expression that captured this information defined in equations (1) and (2) in the main body of the text.

#### Selfing probabilities

In the experiment to explore the effect of mating strategy on heterozygosity, the model was run with input selfing probabilities (*S_u_*) at 0, 0.001 and then from 0.1 to 1 in steps of 0.1. Each experiment at all selfing probabilities described was repeated ten times. A matrix size of 35^2^ was used, with 225 void cells to limit the population to a maximum of 1000 individuals.

#### Pollen and seed dispersal

In the experiment to explore the effect of dispersal on heterozygosity, simulations were run with increasing maximum seed and pollen dispersal distances. To provide sufficient isolation of individuals simulations were run with a matrix size of 135^2^ cells with 17225 void cells. Maximum seed and pollen dispersal were separately increased throughout simulations from 5 to 50 cells in steps of 5 cells. When maximum seed dispersal was increased, maximum pollen dispersal was set to 10 cells, and vice-versa for seed dispersal. With seed dispersal, seeds were given 1000 attempts to find a habitable cell, to avoid population crashes in the simulation. Distance values for pollen dispersal or seed dispersal were then calculated as described above.
